# Innovative therapeutic concepts of progressive multifocal leukoencephalopathy

**DOI:** 10.1007/s00415-021-10952-5

**Published:** 2022-01-07

**Authors:** Nora Möhn, Lea Grote-Levi, Franziska Hopfner, Britta Eiz-Vesper, Britta Maecker-Kolhoff, Clemens Warnke, Kurt-Wolfram Sühs, Mike P. Wattjes, Günter U. Höglinger, Thomas Skripuletz

**Affiliations:** 1 Department of Neurology, Hannover Medical School, Carl-Neuberg-Str. 1, 30625 Hannover, Germany; 2 Institute of Transfusion Medicine and Transplant Engineering, Hannover Medical School, Hannover, Germany; 3 Department of Pediatric Hematology and Oncology, Hannover Medical School, Hannover, Germany; 4 Department of Neurology, University of Cologne, Cologne, Germany; 5 Department of Diagnostic and Interventional Neuroradiology, Hannover Medical School, Hannover, Germany

**Keywords:** Progressive multifocal leukoencephalopathy, Allogeneic virus-specific T cells, Anti-PD-1-antibodies, Immunosuppression, Immune reconstitution

## Abstract

Progressive multifocal leukoencephalopathy (PML) is an opportunistic viral disease of the brain—caused by human polyomavirus 2. It affects patients whose immune system is compromised by a corresponding underlying disease or by drugs. Patients with an underlying lymphoproliferative disease have the worst prognosis with a mortality rate of up to 90%. Several therapeutic strategies have been proposed but failed to show any benefit so far. Therefore, the primary therapeutic strategy aims to reconstitute the impaired immune system to generate an effective endogenous antiviral response. Recently, anti-PD-1 antibodies and application of allogeneic virus-specific T cells demonstrated promising effects on the outcome in individual PML patients. This article aims to provide a detailed overview of the literature with a focus on these two treatment approaches.

**Supplementary Information:**

The online version contains supplementary material available at 10.1007/s00415-021-10952-5.

## Background

### Introductory remarks

**Fig. 1:**
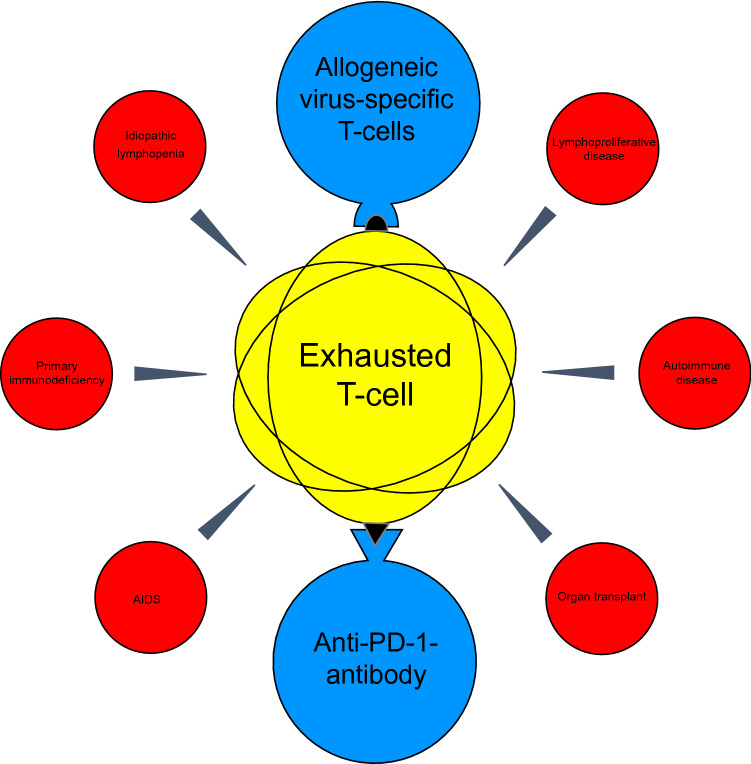
Overview of causes of progressive multifocal leukoencephalopathy and potential innovative treatment options. AIDS, acquired immunodeficiency syndrome; PD-1, programmed cell death protein

Progressive multifocal leukoencephalopathy (PML) is an opportunistic infection of the brain caused by the human polyomavirus 2 (HPyV-2) (previously known as: JC polyomavirus). Overall, PML is associated with severe disability and a relatively high mortality [1]. Infection with HPyV-2 usually occurs during childhood, though the proportion of seropositive persons in the population increases with age, reaching approximately 60–80% in 70-year-olds [2, 3]. The rate is also highly dependent on the serological test applied, with different groups reporting highly variable seropositivity rates. HPyV-2 usually leads to an asymptomatic, lifelong persistent and latent infection in the general population. However, in patients with long-lasting and profound impairment of cellular immunity, HPyV-2 can reactivate from latency or persistent asymptomatic infection and undergo intra-individually acquired viral genomic rearrangements leading to neuroinvasion and lytic infection of white matter (predominantly oligodendrocytes) and neuronal cells in the brain [4, 5]. The in vivo diagnosis of PML is based on the clinical presentation, brain imaging findings (preferably magnetic resonance imaging, MRI) and the detection of the virus in the cerebrospinal fluid (CSF) by polymerase chain reaction (PCR) [6]. The fact that there is no animal model for PML and that HPyV-2 is difficult to grow in culture remains a major challenge for the development of antiviral therapeutic strategies against HPyV-2. To date, direct antiviral therapeutics such as cidofovir, mirtazapine, cytarabine, or mefloquine have failed to improve survival or reduce disability in PML patients [28–31]. Basically, the key to successful treatment of PML is restoring the functions of the immune system (Fig. 1). The aim of this review is to present different therapeutic strategies for the management of PML. In addition to the use of interleukins, the treatment of PML with anti-PD-1 antibodies and allogeneic virus-specific T cells will be characterized in particular. For this purpose, all cases and case series published to date on this topic have been summarized.

### Immunological mechanisms and causative factors of PML

Because most adults are exposed to HPyV-2 in childhood, HPyV-2-specific antibodies, and memory T cells are found in their blood. In patients with a human polyomavirus 1 (HPyV-1) (formerly known as: BK virus) infection, a close relative of HPyV-2 sharing immunologically significant epitopes, it has been shown that an increase in antibody titers is associated with a reduction in viral load [7, 8]. Although these antibodies can effectively control viremia, they cannot control polyomavirus-associated complications. In contrast, the presence of cytotoxic (CD8+) T cells has been shown to correlate positively with a beneficial clinical course of PML [9]. Thus, it is reasonable that an impaired T-cell immune response due to underlying immunosuppressive disease entities or therapy increases the risk of PML. The role of B cells in the pathogenesis of PML is rather unclear. Functional B cells are necessary for adequate viral defense and the risk for PML is quite high, especially in chronic lymphocytic leukemia (CLL) and other B-cell-associated lymphoproliferative disorders. This might be due to the fact that certain B-cell transcription factors (especially Spi-B) promote not only B-cell differentiation but also HPyV-2 replication [10]. In general, the causes of PML can be divided into three major subgroups. The first group consists of patients with human immunodeficiency virus (HIV)-infection whose PML risk is due to the underlying disease alone. At the height of the acquired immunodeficiency syndrome (AIDS) pandemic, approximately 5% of HIV-positive patients developed PML [11]. After the introduction of highly active antiretroviral therapy, both the number of HIV patients developing PML and the mortality of PML in these patients decreased significantly [12]. The risk of developing PML is highly dependent on the number of CD4+ T cells and current overall incidence rate amounts to about 1/1 000 person-years [1, 13–15]. The second group includes patients whose PML risk is due to both their underlying disease and its therapy. Particularly those affected by a lymphoproliferative disease should be mentioned here, since both the disease itself as well as its therapy severely compromises the immune system. To date, PML patients with an underlying malignant hematologic disease have the worst outcome. Mortality in individual studies of smaller size is about 90% and many patients die within the first 2 months after diagnosis [16–19]. The last group comprises patients who receive immunosuppressive therapy, e.g. due to an autoimmune disease. In this context, it should be mentioned that the incidence of monoclonal antibody therapy-related PML has substantially increased in recent years. The best-known example of such an agent certainly is the anti-alfa4-integrin antibody natalizumab, which was designed to prevent the migration of leukocytes into the CNS and is approved for the treatment of highly active relapsing multiple sclerosis. Since its approval in 2004, 839 cases of PML have been reported to date (as of September 2020, www.tysabri.de, accessed 07/10/2021), with risk correlating with duration of treatment and blood HPyV-2 antibody index values (quantifies antibody reactivity relative to a reference sera) [20]. The mortality of natalizumab-related PML is approximately 20% to 23% [21–23], but survivors largely carry severe or at least moderate disability [22]. Prognosis in this group of patients largely depends on the time of diagnosis and the extent of lesion burden on MRI at the time of PML diagnosis. Patients who were not symptomatic when PML is diagnosed and patients with less extensive disease on MRI at diagnosis tend to have a better prognosis with better survival rates and less functional disability [23–25]. A special feature of this group of natalizumab-treated patients is that they are particularly prone to develop a so-called PML-immune reconstitution inflammatory syndrome (PML-IRIS). This phenomenon is characterized by an excessive inflammatory reaction leading to paradoxical worsening after implementing immune system restoration. It can occur in all PML patients in principle, but is particularly well characterized in natalizumab-associated PML [21].

Patients with other autoimmune diseases are also increasingly treated with novel immunomodulatory therapies, especially with monoclonal antibodies such as rituximab or infliximab [26]. The rate of PML cases in these patients is significantly lower than in those with lymphoproliferative diseases. Nevertheless, the risk of PML occurrence must also be considered here [27], especially since the use of such therapies will certainly continue to increase in the future. It should be noted that in this last group of patients, treatment suspension can help in improving PML prognosis while worsening the underlying disease.

### Use of interleukins for the treatment of PML

Several studies described the use of interleukins (interleukin-2 and interleukin-7) in PML to reconstitute the immune response. As a trophic factor for lymphocytes, interleukin-2 (IL-2) is required for the establishment and maintenance of adaptive T-cell responses; however, IL-2 is also critical for immune dysregulation through its effect on regulatory T cells [32]. For example, disruption of IL-2 production after stem cell transplantation appears to lead to a deficiency in cell-mediated immunity and thus to an increased risk of opportunistic infections in stem cell transplanted patients [33]. It is, therefore, not surprising that the positive reports on the use of IL-2 in PML are particularly from patients after stem cell transplantation. In addition, other previously used PML therapies such as cytarabine could not be used because of the risk of cytopenias [34, 35].

There are a total of five case reports on the use of IL-2 in patients with underlying hematologic disease and PML. Two cases received a combination treatment of IL-2 and pembrolizumab. All affected individuals had in common that their underlying disease had led to a partly pronounced lymphocytopenia as the cause of PML. They all benefited from treatment and showed long-term improvement of their neurological symptoms [36–39]. A single publication can be found that addressed IL-2 therapy for natalizumab-associated PML. A 51-year-old female patient with relapsing–remitting multiple sclerosis developed a novel motor aphasia three years after initiation of natalizumab therapy and was diagnosed with PML. Since the symptoms were progressive after plasmapheresis and intravenous immunoglobulins, a therapy with subcutaneous IL-2 was initiated, under which the patient's clinical symptoms improved in the long term and the viral load in the CSF decreased [40].

A total of seven case reports on the use of recombinant interleukin-7 (IL-7) in PML have been published. Each of the patients had (CD4+) lymphopenia as the precipitating cause of PML, either idiopathic (*n* = 3) or due to an underlying disease elsewhere (sarcoidosis (*n* = 1), HIV infection (*n* = 1), hematologic disease (*n* = 2)). IL-7 therapy was administered either intramuscularly or subcutaneously, and in all cases, there was both an increase in CD4+ cells and long-term improvement in neurological symptoms [41–47].

In addition, subcutaneous IL-7 was combined with vaccination against the JCV VP1 protein to target PML. Two patients were treated this way. Both showed an increase in JCV VP1-specific CD4+ T cells and a long-term stabilization or slight improvement of PML-associated symptoms [48]. Another paper by Patel and colleagues described the application of multiple PML therapeutics in a patient with CD4+ lymphopenia. In addition to cidofovir, risperidone, and mefloquine, IL-7 and CMX001, an investigational oral agent, were used [49]. The patient benefited from the treatment, but which therapeutic agent was the possibly decisive one remained unanswered. In summary, the results for the treatment of PML with interleukins are promising especially if PML is based on CD4+ lymphopenia. Since data remain sparse and most case reports are several years old, it is very encouraging that a recent pilot study (NCT04781309) is investigating the value of recombinant IL-7 for the treatment of lymphopenia in PML patients.

Furthermore, in recent years, two modern therapeutic approaches to PML have emerged as promising candidates for the treatment of PML in small case series and single case reports (Table 1). They are both based on the activation of the endogenous immune system and will be characterized in more detail below.

**Table 1:** Overview of published case reports on innovative immunotherapy for progressive multifocal leukoencephalopathy (PML)

	Therapy	Median patient’s age (years)	Underlying diseases	Outcome	Adverse events
Anti-PD-1-antibodies	Nivolumab (*n* = 9)	53 (42–73)	Lymphoproliferative disease (*n* = 6)	4/9 (44%) Improvement of symptoms	PML-IRIS (*n* = 3)
			Idiopathic lymphopenia (*n* = 1)	2/9 (22%) Stabilization of symptoms	Arthritis (*n* = 1)
			Primary immunodeficiency (*n* = 2)	3/9 (33%) Death	Myositis (*n* = 2)
					Parkinsonism (*n* = 1)
					Colitis/Hepatitis (*n* = 1)
	Pembrolizumab (*n* = 21)	68 (31–78)	Lymphoproliferative disease (*n* = 11)	7/21 (33%) Improvement of symptoms	PML-IRIS (*n* = 4)
			AIDS (*n* = 3)	6/21 (29%) Stabilization of symptoms	Exanthema (*n* = 2)
			Idiopathic lymphopenia (*n* = 2)	1/21 (5%) Worsening of symptoms	Psoriasis flare (*n* = 1)
			Primary immunodeficiency (*n* = 3)	7/21 (33%) Death	Diarrhea (*n* = 1)
			Autoimmune disease (*n* = 1)		
			Unknown (*n* = 1)		
Allogeneic virus-specific T cells	HPyV-1-specif. (*n* = 19)	57 (19–73)	Lymphoproliferative disease (*n* = 11)	10/19 (53%) Improvement of symptoms	PML-IRIS (*n* = 2)
			Primary immunodeficiency (*n* = 2)	4/19 (21%) Stabilization of symptoms	
			Autoimmune disease (*n* = 2)	5/19 (26%) Death	
			AIDS (*n* = 1)		
			Tumor disease (*n* = 1)		
			Hepatitis B and D (*n* = 1)		
			Immunodeficiency + hemolytic anemia (*n* = 1)		
	HPyV-2-specif. (*n* = 10)	57 (17–70)	Lymphoproliferative disease (*n* = 9)	3/10 (30%) Improvement of symptoms	PML-IRIS (*n* = 2)
			Idiopathic lymphopenia (*n* = 1)	3/10 (30%) Stabilization of symptoms	
				4/10 (40%) Death	

IRIS, immune reconstitution inflammatory syndrome; PD-1, programmed cell death protein 1

## Anti-PD-1-antibodies

The primary therapeutic scope of anti-programmed death (PD)-1 antibodies such as nivolumab or pembrolizumab are oncological diseases. The pharmacodynamic principle of activating CD8+ T cells to generate a potent anti-tumor response has revolutionized cancer therapy. After the approval for indications such as metastatic malignant melanoma or non-small cell lung cancer, the use of PD-1 antibodies in chronic viral infections has been suggested. Over time, increasing evidence suggested that the PD-1/PD-L1 axis is upregulated during acute viral infections to protect surrounding tissues from an exuberant immune response [50]. However, if the virus cannot be eliminated and a chronic viral infection develops, this mechanism causes exhaustion of the antiviral immune response (so-called exhausted T cells), particularly the CD8+ T cells [50]. The hypothesis that such T-cell exhaustion may also play a role in PML has led to the attempt of using anti-PD-1 antibodies in this chronic viral disease [51]. In addition, increased PD-1 expression was detected not only on T cells in the blood and CSF of PML patients, but also on intralesional T cells in the CNS. Simultaneously, increased PD-L1 expression was shown on macrophages within PML lesions, highlighting the relevance of the PD-1/PD-L1 axis in PML [52]. The first paper on the use of pembrolizumab and nivolumab in PML was published in 2019. Eight PML patients with different underlying diseases (HIV infection (*n* = 2), (non)-Hodgkin's lymphoma (*n* = 4), idiopathic lymphopenia (*n* = 2)) were treated with pembrolizumab at a dose of 2 mg per kg body weight every 4–6 weeks. A maximum of three doses were administered in total. During therapy, two patients experienced improvement of neurological symptoms, stabilization of symptoms occurred in four cases, and two patients did not benefit from therapy. In those patients, worsening of clinical symptoms in combination with increased lesion load on brain MRI and HPyV-2 viral load in CSF was observed [52]. It should be noted that no PML-IRIS occurred in any case. The authors reasoned that this was due to the persistence of lymphopenia in all patients beyond pembrolizumab therapy.

In addition to the eight cases mentioned above, additional 13 individual case reports and two smaller case series (totaling 22 additional patients) were published during the course of the study on the use of PD-1 inhibitors in PML [53–64]. Eight of the 13 individual case reports described the use of pembrolizumab, and the remaining five publications used nivolumab. Clinical improvement was achieved in 7 of 13 cases (54%), 2 patients (15%) stabilized, and 4 (31%) died due to disease progression. For more clinical and demographic details regarding the patients treated see Table S1 (supplementary material).

A recent case series by Roos-Weil and colleagues in 2021 described six PML patients treated with anti-PD-1 antibodies (nivolumab (*n* = 4), pembrolizumab (*n* = 2)). The cause of PML was a hematologic malignancy in four cases, one patient suffered from a primary immunodeficiency, and one case was on immunosuppressive therapy for myasthenia gravis. Within the long-term follow-up 14–33 months after initiation of anti-PD-1 treatment, three of the six patients were still alive, one with clinical improvement and two with stabilization of symptoms. Three patients died despite treatment [65].

We recently published a case series describing three PML patients with underlying hematological diseases. One patient with long-standing Waldenstrom's disease showed marked improvement in his PML-associated symptoms during therapy with pembrolizumab and ultimately became symptom-free, whereas the other two patients suffered a fatal course of PML despite anti-PD-1 therapy. Both had been previously treated with rituximab and had no detectable CD20- and CD19-positive cells in their blood at the time of diagnosis [66].

Formally, no causal conclusions can be drawn from the few publications with a very heterogeneous patient group. What can be concluded, however, is that in addition to the initial positive case reports, there was worsening of PML symptoms and even death of patients despite the use of an anti-PD-1 antibody in 33% and 38% of the cases, respectively (Table 1). The level of HPyV-2 viral load, PD-1 expression of T cells, and the number of HPyV-2-specific CD8+ and CD4+ T cells are discussed as possible factors influencing the success of the treatment [67]. In addition, the T-cell phenotype seems to play an important role. Pawlitzki and colleagues observed that in a PML patient with fatal disease progression receiving pembrolizumab therapy (13th individual case report), the proportion of Ki67+ PD-1+ CD45RA- memory T cells, so-called terminally exhausted T cells, increased significantly [68]. Thus, characterization of immune cell subtypes in PML patients will be of great importance for future therapeutic decisions and prognostic assessments.

In addition, a single case report should be mentioned in which the authors postulated PML as a consequence of therapy with nivolumab. A 54-year-old patient with refractory Hodgkin lymphoma was treated with nivolumab for 13 months after multiple high-dose chemotherapies. In addition, oral steroid therapy for hypocortisolism was ongoing. Thirteen months after initiation of nivolumab therapy, a diagnosis of PML was finally made with increasing neurological deficits, and anti-PD-1 therapy was discontinued due to a presumed causal relationship. Regarding the course, the authors reported that the patient remained alive 5 months after diagnosis, but without relevant improvement in neurological deficits [69]. No other adverse events like this due to nivolumab have been published to date, and it is reasonable to assume that the PML in the aforementioned case was not triggered by anti-PD-1 therapy but was preexisting or due to the underlying malignancy or steroid therapy.

Severe autoimmune phenomena, often been described in oncological patients receiving anti-PD1 therapy [70], have not yet been observed in the treatment of PML. However, in contrast to the initial case series by Cortese et al., PML-IRIS occurred in a total of seven patients in the additional published cases (Table 1). Also, individual less severe autoimmune phenomena, such as single cases of myositis, arthritis or colitis were observed as a consequence of anti-PD-1 therapy. Secondary autoimmune phenomena such as pneumonitis, or hypophysitis are common with anti-PD-1 therapy, and neurologists should be vigilant for evidence of such side effects [71]. Especially in patients with pre-existing autoimmune diseases including multiple sclerosis, therapy with nivolumab or pembrolizumab must be discussed very critically. Considering possible autoimmune side effects, which may also affect the CNS in the form of demyelinating inflammation, anti-PD-1 antibody therapy does not seem to be the right option in certain cases.

## Allogeneic virus-specific T cells

Currently, the most promising therapeutic approach may be the use of allogeneic virus-specific T cells. The method has its origins in hematology and has been mainly used in stem cell transplanted patients with Epstein–Barr virus (EBV), cytomegalovirus (CMV), Adenovirus (HAdV) or HPyV-1 infections [8]. Reactivation of HPyV-2 also plays a role in patients after hematopoietic stem cell transplantation, although PML rates are very low compared with hemorrhagic cystitis due to HPyV-1 [72, 73]. The first treatments involving adoptive transfer of virus-specific T cells occurred in the early 1990s [74–76]. Since then, adoptive T-cell therapy has evolved tremendously. Whereas initially peripheral mononuclear cells from seropositive donors were expanded ex vivo in a time-consuming manner, in the course of time it became possible to isolate virus-specific T cells with major histocompatibility complex (MHC)-multimers in a much more time-effective way [77]. In addition, the risk of graft versus host disease (GVHD) could be minimized by a more targeted selection of virus-specific T cells. Another promising immunotherapeutic approach that has emerged in recent years is the use of HLA-matched T-cell lines from third party donors, which offers the advantage of timely availability of cells for clinical use. The efficacy of this method has been illustrated, particularly for CMV and EBV reactivation after allogeneic stem cell transplantation [78]. In 2011, an Italian group published the case of a young patient with severe chronic GVHD and 5 years of immunosuppressive therapy after allogeneic stem cell transplantation, in whom HPyV-2-specific T cells were used for the first time. At PML diagnosis, the remaining immunosuppression was stopped and antiviral therapy with cidofovir was initiated. In addition, the patient received two infusions of 0.5 and 1.0 × 10^6^ T cells from his stem cell donor, respectively, which had been previously coincubated and activated ex vivo with HPyV-2-specific proteins [79]. After therapy, there was marked improvement in neurological symptoms, a decrease in lesion burden on brain MRI, and lack of detectability of HPyV-2 DNA in CSF. Importantly, there was no evidence of GvHD. However, this positive single case report was not followed by further publications on the use of virus-specific T-cell therapy in PML for several years.

In 2018, the therapy with allogeneic T cells received increased focus as a treatment option for PML. Muftuoglu and colleagues treated three PML patients (32, 35, and 73 years old) with HPyV-1-specific T cells from third party donors, with patients receiving two, three, or four T-cell infusions. Of particular note, two of the three patients suffered from underlying hematological disease (acute myeloid leukemia and polycythaemia vera), which are usually associated with high PML mortality [16–19]. Patient 3 had AIDS due to HIV infection. He had discontinued anti-retroviral therapy 5 years before PML diagnosis because of side effects. All patients experienced a reduction in HPyV-2 viral load in the CSF after the first treatment. With regard to clinical symptoms, two of the three patients experienced a significant reduction or complete remission of neurological symptoms. The third patient, who was 73 years old, experienced stabilization but no improvement of her symptoms and ultimately died under palliative care [80]. The case series suggests that a therapeutic attempt with HPyV-1-specific T cells may be reasonable in PML and probably has an acceptable safety profile, although proof of efficacy cannot be provided.

As HPyV-2 and HPyV-1 share certain significant epitopes (in particular, the capsid protein VP1 and the so-called large T antigen (T-Ag), an important regulatory protein of polyomaviruses), there is cross-reactivity for HPyV-2- and HPyV-1-specific T cells [81]. Since the production of HPyV-1-specific T cells is already established in some manufacturing centers under conditions of "Good Manufacturing Practice (GMP)", their use in PML is obvious.

Encouraged by the work of our colleagues, we also started to treat PML patients with HPyV-1-specific T cells at our center. Recently, the experiences of two successfully treated cases were published [82]. One patient suffered from dermatomyositis as underlying disease, the other patient had developed severe pulmonary fibrosis after successful treatment of Hodgkin's lymphoma, so that she had to undergo lung transplantation with consequent strong immunosuppressive treatment. In comparison to Muftuoglu and colleagues, our clinic does not use preproduced frozen allogeneic peripheral blood mononuclear cells stimulated by HPyV-2 antigens. Rather, we have access to a registry of more than 3500 potential donors. Suitable donors are selected based on the appropriate HLA typing and their T-cell frequency. Direct isolation of antigen-specific T cells is achieved by stimulation with appropriate overlapping peptide mixtures, cytokine capture and magnetic isolation, so that the cells are available after about 16–24 h.

Very recently, the literature on the use of HPyV-1-specific T cells in PML was extended by another case report and a first clinical study. A 57-year-old PML patient with underlying marginal cell lymphoma was initially treated with a total of 10 infusions of pembrolizumab. Because of insufficient reduction of viral load in CSF, the patient additionally received two infusions of HPyV-1-specific T cells at 7-week intervals [83]. During therapy, there was both a significant reduction in viral load and improvement in neurological symptoms.

A first pilot clinical trial about treatment with HPyV-1-specific T cells was presented by Cortese and colleagues in August 2021 [84]. After screening of a total of 26 patients, 12 PML patients were ultimately treated. They received a maximum of three infusions of HPyV-1-specific T cells donated by first-degree relatives. One year after the start of the treatment, seven of the twelve patients were still alive, five patients died of PML. No treatment-associated adverse events were reported. It should be noted that the production time of the final T-cell product in this study amounted to up to 4–6 weeks. Because of the long duration of T-cell production, some individual patients could no longer be included in the study due to symptom exacerbation. The extent to which a longer manufacturing time to the final T-cell product negatively affects patient outcome cannot be conclusively assessed due to limited data.

In addition to HPyV-1 specific therapies, treatment of PML with HPyV-2-specific T cells is also gaining increasing interest. The literature contains a recent single case report and a case series on the use of HPyV-2-specific T cells in PML. In the case of a 59-year-old man with refractory multiple myeloma who had undergone allogeneic stem cell transplantation, HPyV-2-specific T cells were derived from the lymphocytes of the HLA-identical stem cell donor. After termination of the ongoing chemotherapy and subsequent T-cell administration, neurological symptoms stabilized and HPyV-2 was not detected in the cerebrospinal fluid (CSF), whereas there was subtle morphological evidence of an immune reconstitution syndrome. Apart from focal epilepsy secondary to PML, the patient was free of neurological symptoms 12 months after therapy with HPyV-2-specific T cells [85].

In January 2021, an Italian group published a case series on HPyV-2-specific T-cell therapy in nine PML patients, in which cell lines were derived from autologous or allogeneic peripheral blood mononuclear cells by stimulation with protein multimers in a procedure of approximately 4 weeks [86]. Seven of the nine patients suffered from malignant hematologic diseases, six of whom had previously received B-cell depleting therapy (rituximab). Of the nine patients treated, three patients, all of whom had been treated with B-cell depleting therapy as part of their underlying malignant hematologic disease, died as a result of PML. One additional death was attributed to varicella zoster virus (VZV) encephalitis. Two of the surviving patients (each with non-Hodgkin's lymphoma as the underlying disease) had stabilization of neurologic symptoms, and three showed more or less marked improvement of symptoms. In five cases, the HPyV-2-specific cellular immune response was analyzed before and after cell therapy. In four of five patients, there was a relevant increase in interferon-gamma (IFN)-producing HPyV-2-specific T cells after T-cell application, which in turn correlated with a favorable outcome [86].

## Conclusions

PML is a rare but often fatal opportunistic viral disease of the brain, for which there has been no adequate therapeutic strategy yet. Basically, the outcome of patients depends on how quickly the body’s own immune response, which is usually impaired in PML patients, can be restored. The ease with which such immune reconstitution can be achieved depends very much on the underlying disease. Patients with underlying hematological diseases remain particularly problematic, as both the disease itself and its therapy can lead to a significant impairment of the immune system.

The two innovative therapeutic concepts mentioned above show promising results in some cases, although the success of treatment varies considerably between patients, particularly in the case of anti-PD-1 therapy. The therapeutic options for PML described in this article cannot all be applied equally to the different PML subgroups. The primary target group is certainly those patients whose immune response is impaired by the underlying disease and its therapy. In the case of HIV-associated PML, the prognosis can usually be improved with the use of effective antiretroviral therapy alone. If the PML is triggered by an immunosuppressive therapy for the treatment of an autoimmune disease, the termination of this therapy can contribute to the treatment of PML, while at the same time a worsening of the underlying disease can occur. These factors must be considered when selecting a therapeutic regimen for PML. With a low overall number of cases to date, it is not yet possible to draw definitive conclusions regarding the efficacy of the treatment approaches. However, the adverse effects described so far seem to be limited. This is true for anti-PD-1 therapy as well as for the use of virus-specific T cells, although secondary autoimmune phenomena must probably be expected, especially with anti-PD-1 therapy. Based on the literature to date, therapy with allogeneic T cells seems to provide the most promising results in the treatment of PML. The factors for successful therapy, for example the question to what extent a delayed manufacturing time affects the outcome, and whether success can be predicted pre-therapeutically should be the subject of future studies.

## Supplementary Information

Below is the link to the electronic supplementary material.

Supplementary file1 (DOCX 260 kb)
