# Supplementary material for: Innovative therapeutic concepts of progressive multifocal leukoencephalopathy
Source: J Neurol. 2022 Jan 7;269(5):2403–13. doi: 10.1007/s00415-021-10952-5 (PMC8739669; doi:10.1007/s00415-021-10952-5)
Supplement: Supplementary file 1 — Supplementary file1 (DOCX 260 kb) [file 415_2021_10952_MOESM1_ESM.docx]

Supplement, table S1:

Table S1: Clinical characteristics and outcome of treated patients.

AIDS: acquired immune deficiency syndrome, ALL: acute lymphatic leukemia, AML: acute myeloid leukemia, CAR: chimeric antigen receptor, CLL: chronic lymphatic leukemia, cMRI: cerebral magnetic resonance imaging, CSF: cerebrospinal fluid, CTx: chemotherapy, CVID: common variable immunodeficiency, DLBCL: diffuse large B-cell lymphoma, FU: follow-up, GvHD: graft versus host disease, HIV: human immunodeficiency virus, HPyV: human polyomavirus, HSCT: hematopoietic stem-cell transplantation, IL: interleukin, IRIS: immune reconstitution inflammatory syndrome, IVIG: intravenous immunoglobulins, mAB: monoclonal antibodies, MTX: methotrexate, PML: progressive multifocal leukoencephalopathy, SLE: systemic lupus erythematodes, VZV: varicella zoster virus.
